# Protective effect of ethyl acetate fraction from S*emen sojae germinatum*, the processed sprout of Chinese black soybean, on rat experimental osteoarthritis

**DOI:** 10.1186/s12906-020-02920-9

**Published:** 2020-04-19

**Authors:** Jun Wang, Jie Guo, Shulan Li, Mengya Zhang, Bingshu He

**Affiliations:** 1 Hubei Province Key Laboratory of Occupational Hazard Identification and Control, Wuhan University of Science and Technology, Wuhan, 430065 China; 2 New Medicine Innovation and Development Institute, Department of Pharmacy, College of Medicine, Wuhan University of Science and Technology, Wuhan, 430065 China; 3 Department of Orthopedic Surgery, Hubei Provincial Women and Children’s Hospital, Wuhan, 430070 China

**Keywords:** *Semen sojae germinatum*, Chinese black soybean, Ethyl acetate fraction, Osteoarthritis

## Abstract

**Background:**

Our previous in vitro study reported that the ethyl acetate fraction (EAF) of *Semen sojae germinatum* (SSG), the processed sprout of Chinese black soybean, possessed the potent anti-inflammatory and chondroprotective properties. The aim of the present work was to verify the in vivo antiosteoarthritic effect of EAF from SSG on a rat osteoarthritis (OA) model .

**Methods:**

A classical rat OA model was surgically induced by anterior cruciate ligament transaction (ACLT). The OA rats were intra-articularly administered EAF from SSG for 8 weeks. The cartilage and synovial tissues were stained with hematoxylin and eosin (HE) to observe the histopathological changes. Safranin O/fast green staining was used to assess the glycosaminoglycan content in cartilage tissue sections. The expression of type II collagen and matrix metalloproteinase (MMP)-13 in cartilage was measured by immunohistochemistry. The apoptotic chondrocytes in the cartilage sections were detected using TUNEL assay. The concentrations of interleukin (IL)-1β and tumor necrosis factor (TNF)-ɑ in synovial fluid were determined using ELISA.

**Results:**

Intra-articular administration of EAF from SSG well retained the structure and superficial layer of cartilage tissues, ameliorated cartilage lesion and the degradation of cartilage matrix, including proteoglycan and type II collagen, induced by ACLT operation. The ACLT-induced upregulation of MMP-13 expression in the cartilage tissues was resisted by EAF from SSG. Moreover, EAF from SSG inhibited the ACLT-induced chondrocyte apoptosis. Compared to OA model group, the inflammatory status of synovial membrane was improved, the levels of inflammatory cytokines IL-1β and TNF-ɑ in synovial fluid were decreased in rats administrated with EAF from SSG.

**Conclusion:**

These data suggested that EAF from SSG displayed in vivo protective effect on OA development via preventing the degeneration of articular cartilage, inhibiting chondrocyte apoptosis and suppressing synovial inflammation.

## Background

As a common degenerative and painful disease of the load-bearing joints, affecting mainly the knees, osteoarthritis (OA) is characterized by localized loss of articular cartilage, synovial inflammation and compensatory remodeling of adjacent bone [1–3]. In terms of contribution to years of life lived with disease, OA ranks as the 11th greatest contributor and above ischemic heart disease, asthma, Alzheimer’s disease, *etc* [3, 4]. In order to improve the patients’ quality of life, OA treatment focusing on relieving inflammation and pain, improving joint mobility and minimizing the disabling effects of the disease has been intensively explored for decades [2–4]. In the current clinical practice, the most well-accepted and recommended pharmacological treatments for OA are mainly analgesics or anti-inflammatory drugs such as nonsteroidal anti-inflammatory agents, corticosteroid and opioids [3]. However, as is well-known, the long term use of these drugs may cause frequent and severe side effects [4].

In recent years, the antiosteoarthritic activities of some natural components derived from functional foods or medicinal plants have attracted considerable attention because of their efficacy and safety [4]. In our previous studies [5, 6], the potential protective action of *Semen sojae germinatum* (SSG) in OA has been reported. As dry and processed germinating seed of *Glycine max L. Merr.* (*Leguminosae*), a Chinese black soybean variety, SSG has long been used as a single herb improving “knee pain” in traditional Chinese medicine (TCM) for 2000 years. Up to date, SSG is still officially included in the current edition of National Pharmacopoeia of China, and widely applied in TCM clinics in China. In our preliminary study [5], use of SSG as feed in rabbits for 12 weeks significantly delayed the progression of anterior cruciate ligament transaction (ACLT) -induced knee OA. To identify the effective component, we further studied the in vitro anti-inflammatory and pro-proliferative activities of aqueous, petroleum ether, ethyl acetate and n-butanol fractions of SSG in interleukin (IL)-1β-stimulated human OA chondrocytes [5]. The results showed the ethyl acetate fraction (EAF) possessed the most potent anti-inflammatory and chondroprotective properties [5].

In order to verify the protective effect of EAF from SSG on OA development, in the present study, we evaluated the in vivo antiosteoarthritic effect of intra-articular administration of EAF from SSG on a classical rat OA model surgically induced by ACLT. This OA animal model is characterized by joint instability, intra-articular inflammation and structural joint changes, which are similar to typical osteoarthritic features observed in human OA [7, 8]. Our findings would be helpful to uncover the modern pharmacological aspects of SSG, and lay the experimental foundation for the better clinical application or food therapy of SSG for OA control.

## Methods

### Preparation of EAF from SSG

According to our previous studies [5, 6], the EAF was separated from dried SSG, the latter of which was prepared according to the guided processing steps recorded in the National Pharmacopoeia of China. Dried SSG were purchased in May 2017 at the Beijing Tongxintang Co., Ltd. China. The material was identified by Dr. Song Liu who is an expert in the area of pharmacognosy. A voucher specimen was deposited at the herbarium of Department of Pharmacy, College of Medicine, Wuhan University of Science and Technology under number 77910. Then 95% ethanol as the extracting solvent was used to obtain the crude extract from SSG. The crude extract suspended in distilled water was further partitioned with ethyl acetate to obtain the ethylacetate soluble fraction.

### Animals and groups

Female SD rats, weighing 200–220 g, were obtained from Hubei Experimental Animal Research Center (Hubei, China), then housed in conventional polycarbonate cages under controlled temperature (23 ± 3 °C), humidity (55 ± 15%) and photoperiod cycle (12 h light/12 h dark). After a 1-week acclimation period, all animals were randomly divided into 3 groups of 12 rats in each group: (1) sham-operated control group; (2) ACLT OA model control group; (3) ACLT+ intra-articular administation of EAF from SSG group. The anterior cruciate ligaments in the right knees of rats in 2 and 3 groups were surgically transected as previously described [7–9] to establish OA model. Arthrotomy without anterior cruciate ligament damage was performed in sham-operated rats. Four weeks after surgery, the rats in the group 3 received 50 uL of EAF from SSG at the concentration of 1 mg/mL by intra-articular injection once a week for 8 weeks. The sham-operated and OA model groups received an injection of 50 uL saline into the knee joints as control. At 24 h after the last administration, all rats were sacrificed, then the synovial fluid and the right knee joints were collected. In all experiments, the rats were euthanized by cervical dislocation under intraperitoneal anesthesia using 2% pentobarbital sodium at the dose of 60 mg/kg. All experiments were according to the institutional guidelines for the use of laboratory animals, which follow the Chinese Animal Protection Act and National Research Council criteria and animal ethic (PZSHUTCM18101801). The experiment protocols were approved by the Animals Care and Use Committee of Wuhan University of Science and Technology (Approval number: 2018026).

### Histological analysis

The proximal tibias and synovial tissues were dissected from the collected knee joints, fixed with 10% neutral-buffered formalin, subsequently decalcified, dehydrated and embedded in paraffin. The cartilage and synovial tissues were stained with hematoxylin and eosin (HE) to observe the histopathological changes. Safranin O/fast green staining was used to assess the glycosaminoglycan content in cartilage tissue sections. The modified Osteoarthritis Research Society International (OARSI) scoring system [10] was used to score the morphological changes of cartilage and synovial tissues by a pathologist blind to the experimental protocol.

### Immunohistochemistry

The expression of type II collagen and matrix metalloproteinase (MMP)-13 in cartilage was measured by immunohistochemistry. The 5 μm-thick paraffin-embedded cartilage sections were incubated with an anti-type II collagen antibody or an anti-MMP-13 antibody (Servicebio, Wuhan, China), then a corresponding secondary antibody (Servicebio, Wuhan, China). Immune complexes were visualized using 3,3′-diaminobenzidine tetrachloride. Then, sections were counter-stained with hematoxylin.

### Terminal deoxynucleotidyl transferase mediated dUTP nick end labelling (TUNEL) assay

The apoptotic chondrocytes in the cartilage sections were detected using TUNEL assay following the instruction manual of TUNEL Apoptosis Assay Kit (Servicebio, Wuhan, China) .

### Measurement of interleukin (IL)-1β and tumor necrosis factor (TNF)-ɑ levels in synovial fluid

Synovial fluid lavage of rats was collected from the knee joints immediately after sacrifice [9]. The concentrations of IL-1β and TNF-ɑ in synovial fluid were determined using using ELISA kits according to the manufacturer’s instructions (R&D Systems, USA). The urea levels in synovial fluid lavage of each rat were measured using the QuantiChrom Urea Assay Kit (BioAssay Systems, Hayward, CA) to correct the IL-1β and TNF-ɑ values for dilution.

### Statistical analysis

All data were expressed as the mean ± SD, and analyzed using one-way analysis of variance (ANOVA) with post hoc Tukey test by SPSS 22.0 software. *P* < 0.05 or *P* < 0.01 was considered statistically significant.

## Results

### EAF from SSG prevented the degeneration of articular cartilage in ACLT- induced OA rat knee joints

**Fig. 1:**
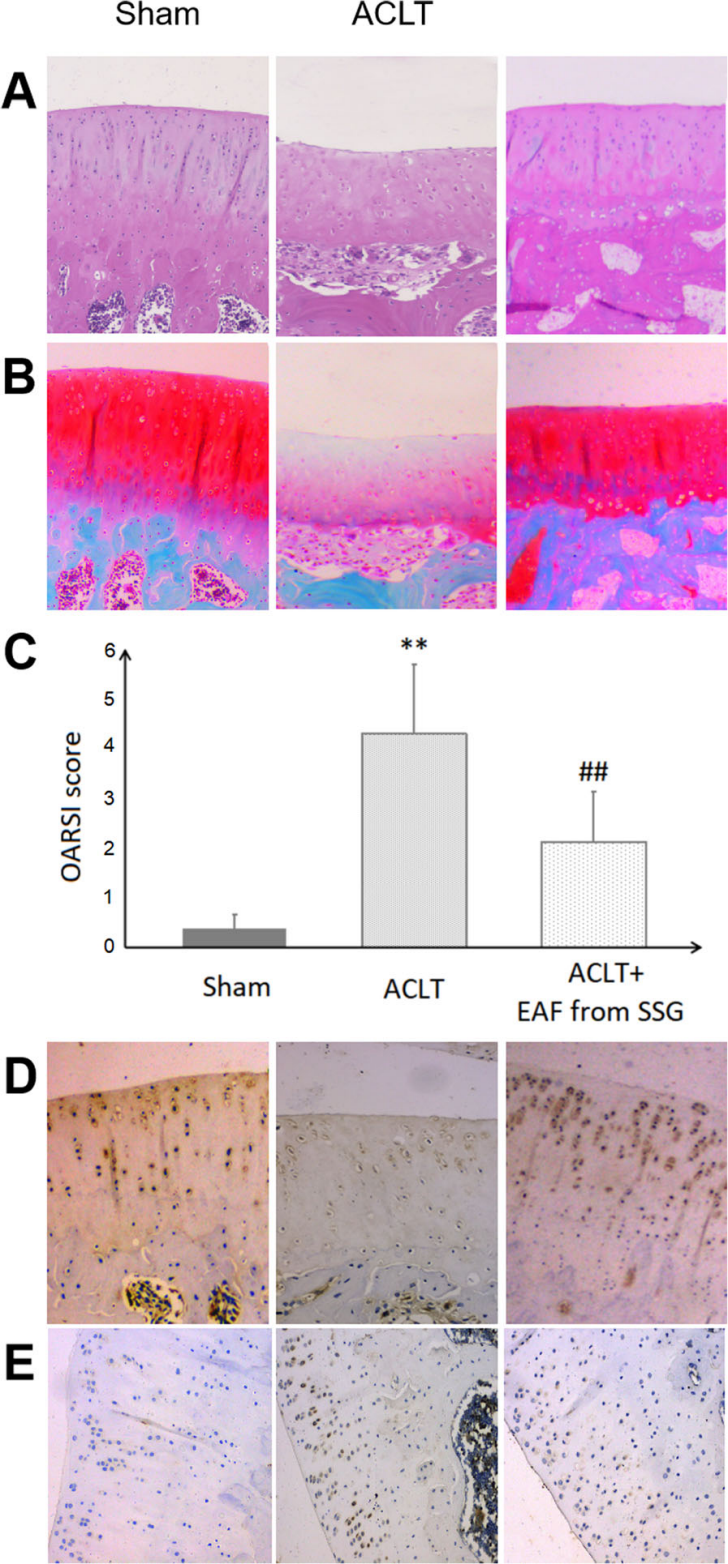
Effect of EAF from SSG on the degeneration of articular cartilage in ACLT-induced OA rat knee joints. **a** Representative images of cartilage sections stained with HE; **b** Representative images of cartilage sections stained with safranin O/fast green; **c** OARSI scores. **d** Representative images of immunohistochemistry staining displaying the expression of type II collagen in cartilage sections. **e** Representative images of immunohistochemistry staining displaying the expression of MMP-13 in cartilage sections. **P<0.01, compared with the sham-operated control group; ##*P*<0.01, compared with the ACLT OA model group.

In order to determine whether intra-articular administration of EAF from SSG, the processed sprout of Chinese black soybean, exerts an in vivo protective effect against the development of rat experimental OA induced by ACLT surgery, the general morphology, proteoglycan and type II collagen contents of articular cartilage from tibial plateau were examined by HE, Safranin-O/fast green and immunohistochemistry staining, respectively. As shown in Fig. 1, in the sham-operated group, the cartilage surface was smooth, the cartilage matrix was normal and the chondrocytes regularly distributed. In the ACLT surgery-induced OA group, the irregular surface of articular cartilage, the thinner cartilage matrix, a remarkable loss of proteoglycan and type II collagen, as well as a decline in chondrocyte number were found, which indicated an articular cartilage injury. However, intra-articular administration of EAF from SSG well retained the structure and superficial layer of cartilage tissues, ameliorated cartilage lesion and matrix degradation induced by ACLT operation. OARSI score of ACLT OA group were significantly higher than that of sham-operated controls (*P* < 0.01). Intra-articular injection of EAF from SSG into the ACLT-induced OA knee joints markedly decreased the OARSI score by 50.2% (*P* < 0.01). These data suggested that EAF from SSG prevented the degradation of cartilage in OA, and stimulated chondrocytes to synthesize proteoglycan and type II collagen.

As one of major protagonists of cartilage degradation during OA progression, MMP-13 plays a pivotal role in proteolysis of type II collagen, and also mediates the breakdown of proteoglycan [11, 12]. Immunohistochemistry showed that intra- articular administration of EAF from SSG resisted the ACLT-induced upregulation of MMP-13 expression in the cartilage tissues, suggesting EAF from SSG prevented cartilage degradation by inhibiting the destructive metalloproteinase MMP-13.

### EAF from SSG protected chondrocytes from apoptosis in ACLT-induced OA rat knee joints

**Fig. 2:**
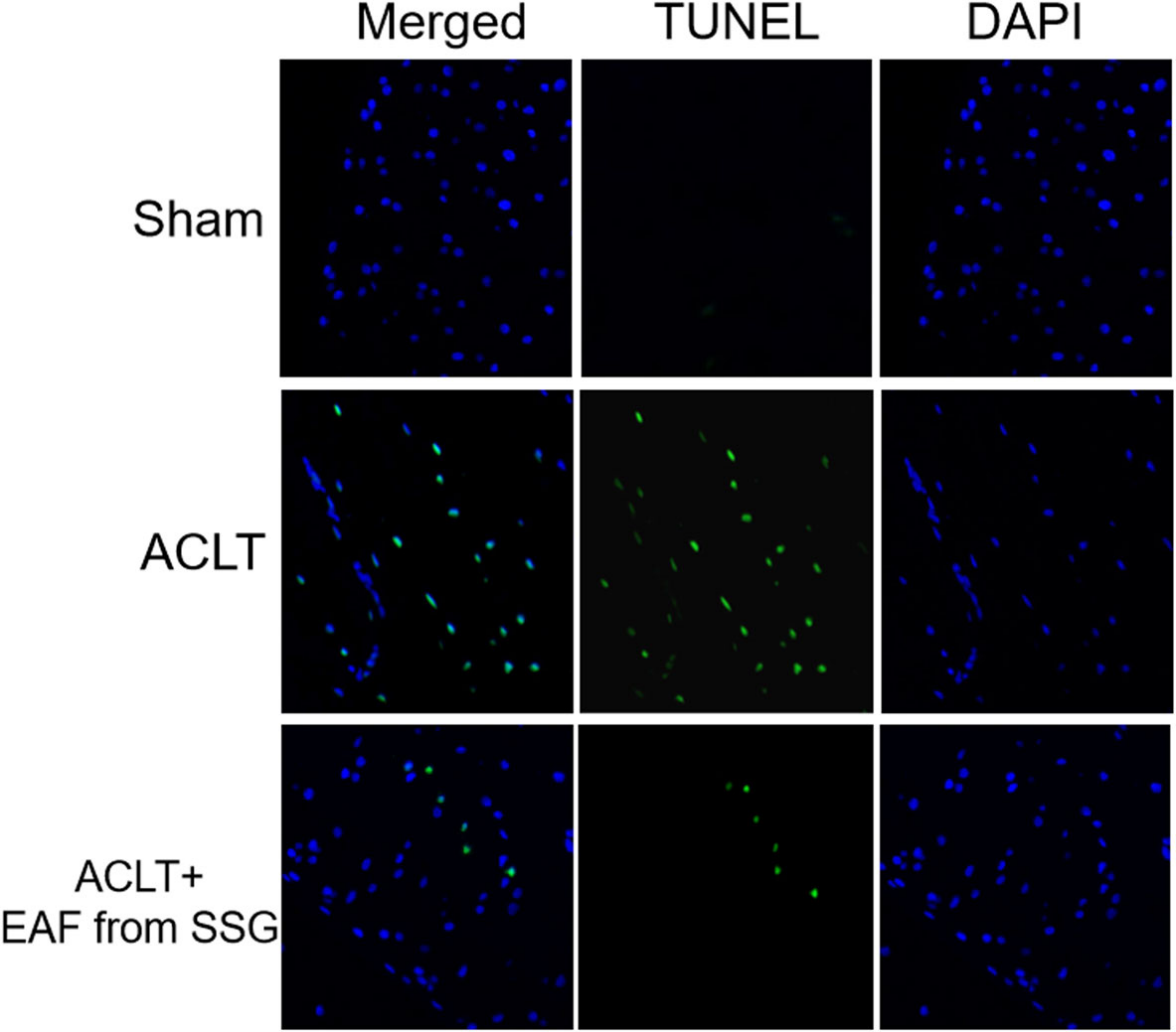
Representative images of TUNEL-stained cartilage tissue sections of rat tibial plateau

As shown in Fig. 2, TUNEL staining was performed in cartilage specimens to evaluate the effects of EAF from SSG on chondrocyte apoptosis. Compared with the sham controls, ACLT operation decreased the total chondrocyte numbers, but a higher proportion of of TUNEL-positive apoptotic chondrocytes was found in ACLT OA group, suggesting the chondrocyte apoptosis in OA knee joints. Importantly, intra-articular administration of EAF from SSG reversed the apoptosis induction effect of ACLT operation on the chondrocytes.

### EAF from SSG suppressed synovial inflammation in ACLT-induced OA rat knee joints

**Fig. 3:**
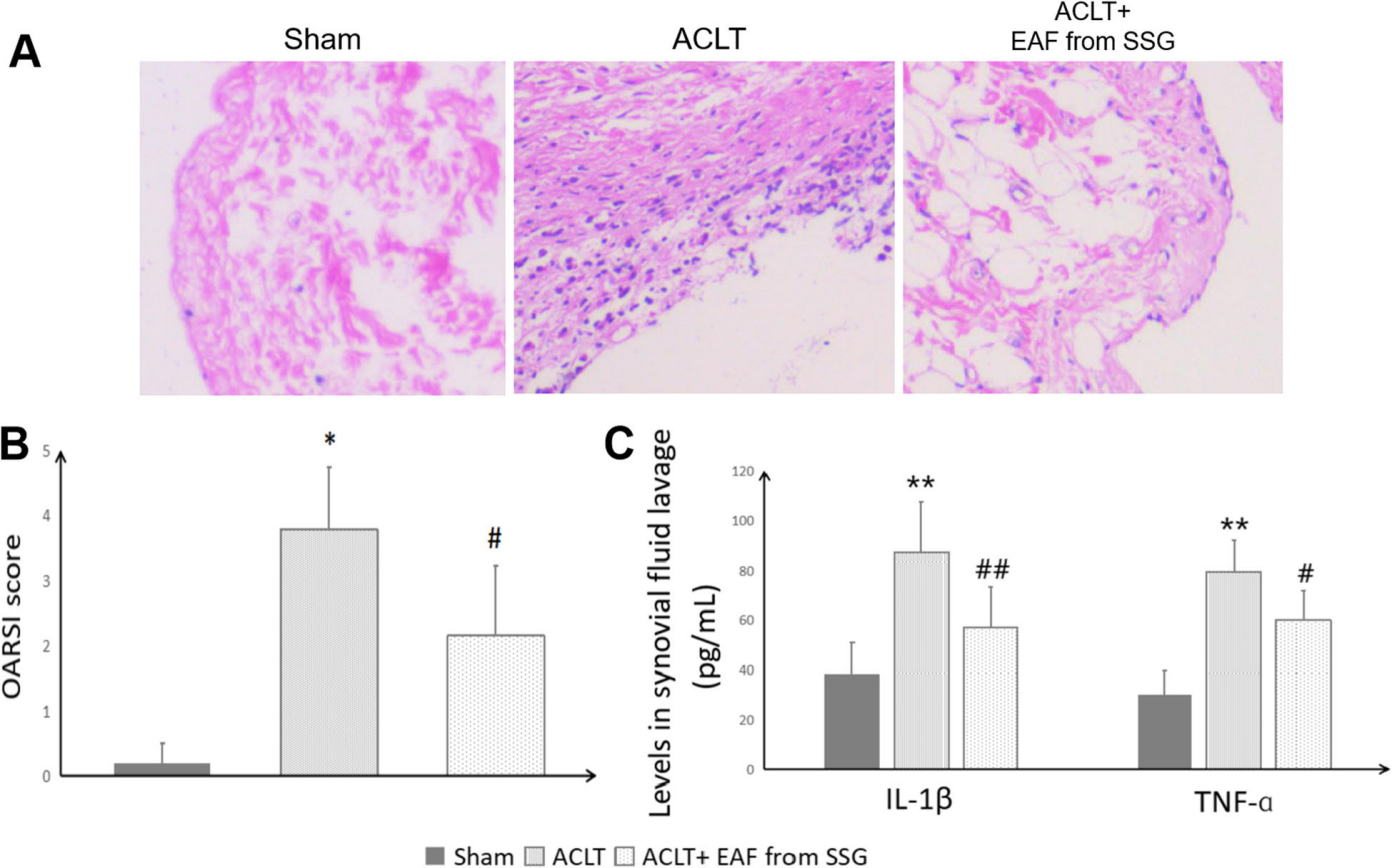
Effect of EAF from SSG on synovial inflammation in ACLT-induced OA rat knee joints. **a** Representative images of HE -stained synovial tissues. **b** OARSI scores of synovial inflammation. **c** The concentrations of IL-1β and TNF-ɑ in synovial lavage. *P < 0.05,**P < 0.01, compared with the sham-operated control group; #*P* < 0.05, ##*P* < 0.01, compared with the ACLT OA model group

Here, synovial inflammation was measured by HE staining on synovial tissue sections, and ELISA assay in the synovial fluid lavage (Fig. 3). HE staining showed synovium in ACLT OA group exhibited inflammatory responses, however, reduced inflammatory cell infiltration and less synovial lining cell layers were found in ACLT+EAF from SSG-administrated rats. Compared with the ACLT OA group, the OARSI scores of synovial inflammation were significantly decreased by intra- articular injection of EAF from SSG (*P* < 0.05).

The inflammatory responses in the synovial membrane further damage the surrounding joint tissues through the release of pro-inflammatory cytokines into the synovial fluid [13]. IL-1β and TNF-ɑ are two important potent inflammatory cytokines contributing to the induction of joint inflammation, cartilage catabolism, even chondrocyte death in OA [14–16]. We found that the urea-adjusted synovial lavage concentrations of IL-1β and TNF-ɑ were significantly enhanced in ACLT OA group, compared to the sham-operated controls (*P* < 0.01). However, the IL-1β and TNF-ɑ contents in the rat synovial lavage were markedly decreased after local administration of EAF from SSG in OA rats (*P* < 0.05; *P* < 0.01). EAF from SSG suppressed ACLT-induced IL-1β and TNF-ɑ secretions from synovium in OA rats by 34.2 and 24.3%, respectively. These data verified the local anti-inflammatory activity of EAF from SSG in OA, which was in line with our previous in vitro observations [6].

## Discussion

At present, it has been clinically recognized that functional dietary food or food products could be used as proper health supplements or phyto-pharmaceuticals to fight against multiple disease conditions [17, 18]. In particular, the benefits of soy-derived products on OA have been clearly established [14–19]. For example, the insufficient consumption of soy products is considered as a predictor for knee OA among elderly Japanese women [19], while soy intake improved the biochemical markers and the symptoms of OA [20]. A Chinese cross-sectional study conducted by Li et al. [18] also showed that dietary intake of soy milk, a stable emulsion of water,oil, and soy protein, was negatively associated with the osteophyte formation in knee joint. The effectiveness and safety of soy unsaponifiables, as symptomatic slow acting agents for OA, have been demonstrated in clinical studies of knee OA [21]. As an isoflavone in soybeans and soy products, genistein could suppress the production of proinflammatory molecules and increase the synthesis of glycosaminoglycans in chondrocytes [22]. In addition, daidzin as another isoflavone compound used for the quality control of SSG [5] has been shown to possess the anti-inflammatory activity in rat hemorrhagic cystitis [23] and dry eye rat model [24].

Our previous studies [5] demonstrated SSG, the processed sprout of Chinese black soybean used as a traditional Chinese medicine for knee pain, may exert disease-modification effect in OA and be a safe alternative to current OA therapies. Based on the results from an in vitro screening assay to detect the potential antiosteoarthritic fractions of SSG produced by solvent-fractionation [6], this study further showed the EAF from SSG contains bioactive constituents that have in vivo protective effect on the development of OA.

EAF from SSG well preserved the structure of cartilage tissues and the integrity of cartilage matrix in the ACLT-induced OA knee joints. As a defining feature of OA, the degradation of cartilage matrix, including the proteoglycan and type II collagen, leads to gradual loss of cartilage from the surface of joints [11]. Proteoglycan and type II collagen are well-accpeted as two key structural elements in the articular cartilage [11]. Proteoglycan provides the water-holding ability of cartilage, thus protects the weight-bearing joints under high compressive loads [11]. As most abundant collagen in cartilage matrix, type II collagen accounts for 95% of the total collagen content in cartilage [11, 23]. Type II collagen scaffolds impart elastic restraint essential for maintaining the cartilage integrity [25]. In this study, the proteoglycan and type II collagen contents in OA cartilage examined by Safranin-O/fast green and immunohistochemistry staining, respectively, were significantly enhanced by the administration of EAF from SSG. The mechanism underlying this regulation of cartilage matrix production might be due to the inhibition of the destructive metalloproteinase such as MMP-13 by EAF from SSG.

As the dominant cell population present in articular cartilage, chondrocytes play an important role in the cartilage metabolic homeostasis and are crucial for joint function. Chondrocyte apoptosis is a crucial mechanism involved in the pathology of OA. As a form of programmed cell death, apoptosis of chondrocytes leading to their gradual loss has been shown to be associated to the initiation and severity of cartilage destruction as well as matrix depletion in OA tissues [26–28]. Due to the limited regeneration capacity of the cartilage, the inhibition of chondrocyte apoptosis provides a critical pathway for the effective preservation of chondrocytes in cartilage, thus has been believed as an important strategy for OA control [28]. EAF from SSG protected the chondrocytes from apoptosis in OA knee joints, which might also be an important mechanism underlying antiosteoarthritic effect of EAF from SSG.

Although the exact pathogenesis of OA is still unsure, the altered inflammatory state has been proposed as the underlying cause of OA, and the mechanical stress as the inducer [3, 29]. As a hallmark feature at multiple stages of OA, synovial inflammation is a result of interactions between the joint damage factors and immune system, contributes to early OA symptoms and later structural deterioration, thus has been considered as a key risk factor in the initiation and progression of OA disease [13, 30]. Accordingly, anti-inflammatory therapeutics has great potential to delay structural alterations and control symptoms, especially in early stage of OA [30]. The anti-inflammatory properties of several soy products such as soy unsaponifiables [15], genistein [22] and daidzin [23, 24] have been demonstrated. In the present study, we found EAF from SSG improved the pathological inflammation and suppressed IL-1β and TNF-ɑ secretions from synovium in ACLT-induced OA rats.

There is a limitation in this study that the female rats used here were not in the control of estrous cycle stage. So the possibility that the estrous cycle of rats affects the anti-inflammatory response cannot be completely ruled out. However, because the majority of rats were synchronized in the breeding house, the observed differences in our study are probably not due to hormonal differences among the groups.

## Conclusion

This study demonstrated the antiosteoarthritic effect of EAF from SSG, the later of which is the processed sprout of Chinese black soybean, on a rat OA model surgically induced by ACLT. Intra-articular administration of EAF from SSG in ACLT-operated rat osteoarthritic knees significantly ameliorated the histological lesion and matrix degradation in articular cartilage, improved the chondrocyte apoptosis and inflammatory cell infiltration in synovial membrane, and decreased the enhanced IL-1β and TNF-ɑ contents in synovial lavage. These findings suggested that EAF from SSG might be a possible choice for treating OA.

## Data Availability

The datasets used and/or analysed during the current study available from the corresponding author on reasonable request.

## Abbreviations

ANOVA: Analysis of variance
EAF: Ethyl acetate fraction
ELISA: Enzyme-linked immunosorbent assay
HE: Hematoxylin and eosin
IL: Interleukin
MMP: Matrix metalloproteinase
OA: Osteoarthritis
OARSI scoring: Osteoarthritis Research Society International scoring
SSG: *Semen sojae germinatum*
TCM: Traditional Chinese medicine
TNF: Tumor necrosis factor
TUNEL: Terminal deoxynucleotidyl transferase mediated dUTP nick end labelling

## References

[1] Mandl LA (2018). Osteoarthritis year in review 2018: clinical. Osteoarthr Cartil.

[2] Majeed MH, Sherazi SAA, Bacon D, Bajwa ZH (2018). Pharmacological treatment of pain in osteoarthritis: a descriptive review. Curr Rheumatol Rep.

[3] Chin KY (2016). The spice for joint inflammation: anti-inflammatory role of curcumin in treating osteoarthritis. Drug Des Devel Ther.

[4] Henrotin Y, Mobasheri A (2018). Natural products for promoting joint health and managing osteoarthritis. Curr Rheumatol Rep.

[5] He B, Wang J (2013). Effect of *semen sojae germinatum* on experimental osteoarthritis in the rabbit knee. Bangl J Pharmacol.

[6] Fan W, Qian Y, Wang J, Yang X, Gui T, He B (2016). Chondroprotective and anti-inflammatory activities of extracts from S*emen Sojae Germinatum* on IL-1β- stimulated human osteoarthritis chondrocytes. Indian J Pharm Edu.

[7] Murata K, Kanemura N, Kokubun T, Fujino T, Morishita Y, Onitsuka K, Fujiwara S, Nakajima A, Shimizu D, Takayanagi K (2017). Controlling joint instability delays the degeneration of articular cartilage in a rat model. Osteoarthr Cartil.

[8] Thomas NP, Wu WJ, Fleming BC, Wei F, Chen Q, Wei L (2017). Synovial inflammation plays a greater role in post-traumatic osteoarthritis compared to idiopathicosteoarthritis in the Hartley Guinea pig knee. BMC Musculoskelet Disord.

[9] Zhang Y, Wei X, Browning S, Scuderi G, Hanna LS, Wei L (2017). Targeted designed variants of alpha-2-macroglobulin (A2M) attenuate cartilage degeneration in a rat model of osteoarthritis induced by anterior cruciate ligament transection. Arthritis Res Ther.

[10] Gerwin N, Bendele AM, Glasson S, Carlson CS (2010). The OARSI histopathology initiative -recommendations for histological assessments of osteoarthritis in the rat. Osteoarthr Cartil.

[11] Becerra J, Andrades JA, Guerado E, Zamora-Navas P, López-Puertas JM, Reddi AH (2010). Articular cartilage: structure and regeneration. Tissue Eng Part B Rev.

[12] Wan Y, Li W, Liao Z, Yan M, Chen X, Tang Z. Selective MMP-13 inhibitors: promising agents for the therapy of Osteoarthritis. Curr Med Chem. 2018. 10.2174/0929867326666181217153118. 10.2174/0929867326666181217153118 30556497

[13] Wang X, Hunter DJ, Jin X, Ding C (2018). The importance of synovial inflammation in osteoarthritis: current evidence from imagingassessments and clinical trials. Osteoarthr Cartil.

[14] Sumi C, Hirose N, Yanoshita M, Takano M, Nishiyama S, Okamoto Y, Asakawa Y, Tanimoto K (2018). Semaphorin 3A inhibits inflammation in chondrocytes under excessive mechanical stress. Mediat Inflamm.

[15] Charlier E, Relic B, Deroyer C, Malaise O, Neuville S, Collée J, Malaise MG, De 468 Seny D. Insights on Molecular Mechanisms of Chondrocytes Death in 469 Osteoarthritis. Int J Mol Sci. 2016;17(12). 10.3390/ijms17122146 PMC5187946 27999417

[16] Cheng X, Li K, Xu S, Li P, Yan Y, Wang G, Berman Z, Guo R, Liang J, Traore S, Yang X (2018). Applying chlorogenic acid in an alginate scaffold of chondrocytes can improve the repair of damaged articular cartilage. PLoS One.

[17] Choudhary D, Kothari P, Tripathi AK, Singh S, Adhikary S, Ahmad N, Kumar S, Dev K, Mishra VK, Shukla S, Maurya R, Mishra PR, Trivedi R (2018). Spinacia oleracea extract attenuates disease progression and sub-chondral bone changes in monosodium iodoacetate-induced osteoarthritis in rats. BMC Complement Altern Med.

[18] Li H, Zeng C, Wei J, Yang T, Gao SG, Li YS, Luo W, Xiao WF, Xiong YL, Lei GH (2016). Relationship between soy milk intake and radiographic knee joint space narrowing and osteophytes. Rheumatol Int.

[19] Kojima N, Kim M, Saito K, Yoshida Y, Hirano H, Obuchi S, Shimada H, Suzuki T, Kim H (2017). Predictors of self-reported knee osteoarthritis in community-dwelling older women in Japan: a cross-sectional and longitudinal cohort study. Arch Gerontol Geriatr.

[20] Arjmandi BH, Khalil DA, Lucas EA, Smith BJ, Sinichi N, Hodges SB, Juma S, Munson ME, Payton ME, Tivis RD, Svanborg A (2004). Soy protein may alleviate osteoarthritis symptoms. Phytomedicine..

[21] Al-Afify ASA, El-Akabawy G, El-Sherif NM, El-Safty FEA, El-Habiby MM (2018). Avocado soybean unsaponifiables ameliorates cartilage and subchondral bone degeneration in mono-iodoacetate-induced knee osteoarthritis in rats. Tissue Cell.

[22] Shen CL, Smith BJ, Lo DF, Chyu MC, Dunn DM, Chen CH, Kwun IS (2012). Dietary polyphenols and mechanisms of osteoarthritis. J Nutr Biochem.

[23] Wu KC, Lin WY, Sung YT, Wu WY, Cheng YH, Chen TS, Chiang BJ, Chien CT (2019). Glycine tomentella hayata extract and its ingredient daidzin ameliorate cyclophosphamide-induced hemorrhagic cystitis and oxidative stress through the action of antioxidation, anti-fibrosis, and anti-inflammation. Chin J Phys.

[24] Xiao F, Cui H, Zhong X (2018). Beneficial effect of daidzin in dry eye rat model through the suppression of inflammation and oxidative stress in the cornea. Saudi J Biol Sci.

[25] Noé B, Poole AR, Mort JS, Richard H, Beauchamp G (2017). Laverty S.C2K77 ELISA detects cleavage of type II collagen by cathepsin K in equine articular cartilage. Osteoarthritis Cartilage.

[26] Gu YT, Chen J, Meng ZL, Ge WY, Bian YY, Cheng SW, Xing CK, Yao JL, Fu J, Peng L (2017). Research progress on osteoarthritis treatment mechanisms. Biomed Pharmacother.

[27] Hwang HS, Kim HA (2015). Chondrocyte apoptosis in the pathogenesis of osteoarthritis. Int J Mol Sci.

[28] Pountos I, Giannoudis PV (2017). Modulation of cartilage's response to injury: can chondrocyte apoptosis be reversed?. Injury.

[29] Mora JC, Przkora R, Cruz-Almeida Y (2018). Knee osteoarthritis: pathophysiology and current treatment modalities. J Pain Res.

[30] Robinson WH, Lepus CM, Wang Q, Raghu H, Mao R, Lindstrom TM, Sokolove J (2016). Low-grade inflammation as a key mediator of the pathogenesis of osteoarthritis. Nat Rev Rheumatol.

